# Influence of C-Peptide on Glucose Utilisation

**DOI:** 10.1155/2008/769483

**Published:** 2008-03-27

**Authors:** B. Wilhelm, P. Kann, A. Pfützner

**Affiliations:** ^1^Institute for Clinical Research and Development, Parcusstraße 8, Mainz 55116, Germany; ^2^Department of Endocrinology and Diabetes, Philipps University, Marburg 35032, Germany

## Abstract

During the recent years, multiple studies demonstrated that C-peptide is not an inert peptide, but exerts important physiological effects. C-peptide binds to cell membranes, stimulates the Na,K-ATPase and the endothelial nitric oxide (NO) synthase. Moreover, there is evidence that C-peptide decreases glomerular hyperfiltration and increases glucose utilisation. Nevertheless, there is still limited knowledge concerning mechanisms leading to an increased glucose utilisation either in rats or in humans. The aim of this paper is to give an overview over the published studies regarding C-peptide and glucose metabolism from in vitro studies to longer lasting studies in humans.

## 1. IN VITRO STUDIES

Zierath et al. [1] showed that physiological concentrations of human C-peptide stimulate glucose transport in human skeletal muscle in a dose-dependent manner. In order to elucidate the mechanisms by which C-peptide stimulates glucose transport in human skeletal muscle, they investigated the interaction between C-peptide and insulin binding to receptors, the potential role of C-peptide in activating the receptor tyrosine kinase, the influence of counter-regulatory hormones on the C-peptide activation of the glucose transport, and the effect of C-peptide in glucose transportation in skeletal muscle from patients with insulin-dependent diabetes mellitus [2]. They demonstrated that C-peptide partly shares a common pathway with insulin in stimulating skeletal muscle glucose transportation as simultaneous exposure of maximal concentrations of insulin and C-peptide did not result in an additive effect of 3-o-methylglucose transport. C-peptide did not alter the binding of insulin to the insulin receptor nor was shown to have a specific binding to muscle crude membranes. C-peptide stimulates glucose transport by mechanism independent of insulin receptor and tyrosine kinase activity and in contrast to insulin, catecholamines did not reveal a counter-regulatory effect on the C-peptide mediated glucose transport. Under in vitro conditions, a stimulation of glucose transportation could be also demonstrated by patients with insulin-dependent diabetes mellitus. Despite chronic deprivation of C-peptide in vitro, exposure of skeletal muscle strips from patients with insulin-dependent diabetes mellitus to a physiological concentration of C-peptide increased the glucose utilisation measured by an augmented 3-o-methylglucose transport [2]. In vitro studies with isolated mouse muscle showed that C-peptide did not stimulate glycogen synthesis in isolated mouse muscle [3].

## 2. ANIMAL STUDIES

In 1983 it has been demonstrated that in alloxan-treated rats, supra-physiololgical concentrations of C-peptide increased and prolonged the hypoglygemic effect of exogenous insulin on whole body glucose uptake [4]. As glucose transport can be stimulated by NO [5, 6], the aim of an euglycemic clamp study in streptozotocin-induced diabetes rats was to examine whether C-peptide in physiological concentrations increases whole body glucose utilisation and whether such an effect is diminished by an NO synthase inhibitor. Physiological concentrations of homologous rat C-peptide I or II augmented significantly glucose disposal rate (GDR) by 80–90% and metabolic clearance rate (MCR) for glucose by 100–125% in diabetic rats, whereas no effects could be detected in healthy control rats. A further increase in C-peptide concentrations did not let to further effects on GDR or MCR [7]. L-NMMA, a known inhibitor of NO-synthase, was able to block about 85% of the C-peptide-induced increase in GDR. These results suggest that a major proportion of the C-peptide stimulation of glucose utilisation is mediated by NO [7].

Moreover, it could be shown that C-terminal fragments, but not fragments from the middle segment of C-peptide, are as effective as the full-length peptide in stimulating whole-body glucose turnover in streptozotocin-induced diabetes rats [8].

## 3. SHORT-TERM HUMAN STUDIES

During a euglygemic clamp study in 11 patients with type 1 diabetes mellitus C-peptide was infused in two periods over 60 minutes in a concentration of 5 and 30 pmol/kg/min and compared with 10 patients who received saline infusion. After infusion of low-dose C-peptide, whole body glucose utilisation rose by approximately 25% (*P* < .05), whereas no changes in the saline group were detected. The high-dose C-peptide infusion given to 7 of the 11 patients resulted in a small further increase of about 15% (*P* < .05) in glucose utilisation [9].

**Figure 1:**
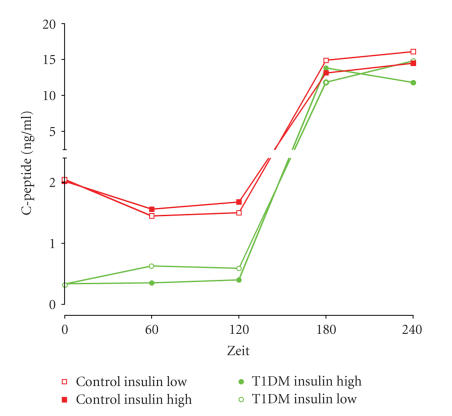
C-peptide levels in healthy controls and patients with diabetes type 1 during the euglycemic clamp.

**Figure 2:**
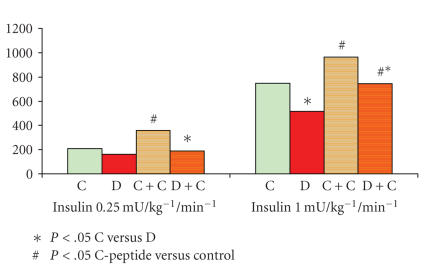
Glucose utilisation in g during an euglygemic clamp with low and high insulin concentrations in healthy controls (C), Diabetes mellitus type 1 and after administration of C-peptide (C+C) and (D+C).

Forearm uptake of glucose after a 5-minute rhythmic dynamic exercise with a handergometer increased significantly after C-Peptide infusion (−4.8 ± 3.1 versus 13.6 ± 3.2 umol · min^−1^100 mL^−1^) in patients with diabetes mellitus [10]. We investigated in our study with 13 patients with type 1 diabetes mellitus and 13 healthy control glucose utilisation after administration of C-peptide (8 pgmol/kg/body weight/min) over 2 hours during an euglycemic clamp procedure with either a high-dose (1.0 Ul/kg body weight/min) or a low-dose insulin infusion (0.25 mU/kg body weight/min). The C-peptide levels reached are shown in Figure 1. After C-peptide infusion, glucose utilisation increased in patients with diabetes mellitus (51.5 ± 25.6 versus 74.51 ± 22.93 g) and healthy controls (74.91 ± 22.01 versus 99.38 ± 24.24 g) statistically significant (*P* < .001) during high-dose insulin infusion and from 16.31 ± 13.34 to 18.8 ± 16.2 g in the diabetic patients and from 20.74 ± 9.96 to 35.8 ± 13.5 g in the healthy controls, the results are given in Figure 2 [11]. In a recent study [12], it has been discussed that C-peptide might increase the bioavailability of insulin by promoting the disaggregation of hexameric insulin. The combined injection of insulin and C-peptide required a greater amount and longer duration of glucose than insulin alone in patients with diabetes type 1. In another setting, the same group applied insulin and C-peptide in the same and in two separate depots and found that the reduction in plasma glucose was significantly faster when administered in the same depot. The amount of glucose that has to be infused in order to avoid a hypoglycaemia was 129% (*P* < .01) by administration in the same depot.

## 4. LONG-TERM HUMAN STUDIES

In a randomized, double-blind study 18 patients with type 1 diabetes received either regular insulin mixed with equimolar amounts of biosynthetic human C-peptide or insulin alone for 1 month as subcutaneous infusions using an insulin pump. At the end of the study, fructosamine levels decreased by about 16% from 3.8 ± 0.3 to 3.2 ± 0.1 (*P* < .05) mmol/L and HbA1c by about 10% from 8.0 ± 0.7% to 7.3 ± 0.5% (*P* < .05%). Fasting blood glucose tended to be lower in the insulin and C-peptide treated group (NS). No statistically significant changes could be demonstrated in the insulin group [13].

## 5. DISCUSSION

C-peptide in nanomolar concentrations binds specifically to cell membranes, assumable by a G-protein-coupled receptor. After activation of a Ca (2+)- and MAP Kinase-dependent pathway, the Na,K-ATPase and the endothelial nitric oxide synthase are stimulated, resulting in an increase of nitric oxide [14]. Increased local release of nitric oxide by C-peptide, resulting in an increased subcutaneous blood flow, might enhance insulin absorption and therefore glucose utilisation [15].

Several studies as cited above have shown that C-peptide increases glucose utilisation either in vitro or in vivo. The effect seems to be a consequence of the stimulation of glucose transport in the skeletal muscle, independent of the insulin receptor or the thyrosine kinase activity, but mediated through nitric oxide [7]. When C-peptide concentrations are increased above the physiological range, no further stimulation of glucose utilisation can be demonstrated [7]. This might be explained by the hypothesis that C-peptide receptors on cell membranes are relatively few and show high-affinity binding, thereby reaching saturation at low C-peptide concentrations [15].

In conclusion, recent studies have demonstrated that C-peptide is not an inert peptide, but a biologically active substance which has besides other effects regulatory influence on glucose metabolism. But still many mechanisms of C-peptide action have to be resolved.

Longer lasting studies are needed in order to evaluate continuing improvement of glucose utilisation in patients with type 1 diabetes.
